# Effect of thymoquinone on NRF2/NF-kB/MAPK pathway in methotrexate-induced rat testis injury

**DOI:** 10.22038/ijbms.2024.77714.16813

**Published:** 2024

**Authors:** Emin Kaymak, Tayfun Ceylan, Tuğrul Akın, Nurhan Kuloğlu, Meryem Sayan, Necla Değer, Esra Önal, Aysegul Burcin Yildirim, Derya Karabulut

**Affiliations:** 1 Department of Histology and Embryology, Faculty of Medicine, Yozgat Bozok University, Yozgat, Turkey; 2 Cappadocia University, Department of Medical Services and Tecnigue, Pathology Laboratory Techniques, Nevşehir, Turkey; 3 İstinye University, Department of Medical Biology, İstanbul, Turkey; 4 Niğde Ömer HalisdemirUniversity, Niğde Zübeyde Hanım Vocational School Of Health Services, Niğde, Turkey; 5 Department of Histology and Embryology, Erciyes University Institute of Health Sciences, Erciyes University, Kayseri, Turkey; 6 Gaziantep Islam Science and Technology University, Department of Histology and Embryology, Gaziantep, Turkey; 7 Department of Histology and Embryology, Faculty of Medicine, Erciyes University, Kayseri, Turkey

**Keywords:** Inflammation, Methotrexate, Oxidative stress, Testis, Thymoquinone

## Abstract

**Objective(s):**

In this study, we aimed to investigate the protective effect of Thymoquinone (THQ) against testicular damage caused by Methotrexate (MTX).

**Materials and Methods:**

This study consists of 5 groups: Control, Olive oil, THQ, MTX, and MTX+THQ. At the end of the experiment, spermiogram analysis was performed on the rats. In addition, testicular tissues were taken and histopathology, immunohistochemistry, and biochemistry analysis were performed. Biochemical analyses were performed on the serums.

**Results:**

According to the results obtained, spermiogram values, Johnson’s testicular biopsy score, SOD, CAT, GPx, FSH, LH, and testosterone values were statistically significantly decreased in the MTX group compared to the control group. In the MTX+THQ group, spermiogram values, Johnson’s testicular biopsy score, SOD, CAT, GPx, FSH, LH, and testosterone values increased statistically significantly compared to the MTX group. NRF2 and HO-1 immunoreactivity were statistically significantly decreased in the MTX group compared to the control group. In the MTX+THQ group, NRF2 and HO-1 immunoreactivity were statistically significantly increased compared to the MTX group. The level of MDA, which is important in lipid damage, and the level of biochemistry results of TNF-α, IL1-β, and IL-6, which are important markers, and the results of p-NF-kB and P38 immunoreactivity were statistically significantly increased in the MTX group compared to the control group. In the MTX+THQ group, these parameters showed a significant decrease compared to the MTX group.

**Conclusion:**

According to these results, it is thought that THQ will play a protective role against infertility caused by chemotherapy-induced testicular damage.

## Introduction

A cornerstone in the development of today’s cancer therapy, chemotherapy is one of the most effective and powerful strategies for treating malignant tumors.

However, chemotherapeutic drugs cannot selectively target tumor cells. Almost all anticancer agents have serious side effects on normal tissues and organs (1). Methotrexate [MTX], a folate antagonist compound, is widely used in the treatment of rheumatoid arthritis [RA], psoriasis, breast cancer, and osteosarcoma (2-5). Besides the benefits of MTX, there are also serious side effects. These side effects include kidney, liver, heart, ovary, and testicular damage (6-10). Because of its inhibitory effect on MTX DNA synthesis, repair, and cellular replication, it affects healthy tissues and cells and causes functional disorders in both somatic and reproductive cells (11, 12). It has been observed that MTX has a cytotoxic effect on spermatogenic cells, causes damage to the seminiferous tubule, and causes a significant decrease in sperm count and sperm motility (13-15). Oxidative stress plays an essential role in MTX-induced testicular damage (16).

Studies have shown that MTX reduces the effectiveness of the antioxidant enzyme system and thus causes damage and sensitizes cells to reactive oxygen particles (17). Oxidative stress, which causes testicular damage, develops due to the increase in reactive oxygen species (18). Therefore, antioxidants play an important role in reducing tissue damage against the harmful effects of oxidative stress (16, 19). Natural products play an important role in the treatment of many diseases. Herbal medicines have attracted attention, especially in recent years. Thymoquinone, an active component of Nigella sativa seed oil, is used as an antioxidant, anti-inflammatory, and antitumor agent. Therefore, researchers are important for exploring the molecular mechanisms and potential clinical use of these properties (20-22). Its low systemic toxicity and high biological activity make it a promising alternative to conventional therapeutic drugs (23). Today, THQ is recognized as a powerful antioxidant in the treatment of many injuries.

The oxidation/antioxidant balance is inhibited by external stimuli, thereby increasing the level of reactive oxygen species [ROS]. Excessive ROS production can lead to oxidative damage, alteration of membrane structure and function, and lipid peroxidation (24, 25). Nrf2 [nuclear factor erythroid 2 associated factor 2] is a known redox-sensitive transcription factor that plays a vital role in preventing oxidative stress by maintaining intracellular redox states (26). Heme oxygenase-1 [HO-1] plays an important role in maintaining cellular homeostasis, and increasing HO-1 expression is a protective mechanism against oxidative stress (27). Nrf2 is released and transmits the stress signal to the nucleus for the activation of a specific set of genes encoding phase II antioxidant enzymes, as well as stress-sensitive proteins such as HO-1. They act in coordination with Nrf2 and HO-1, and HO-1 is an Nrf2 target gene that is central to Nrf2-mediated inhibition of Nuclear factor kappa B (NF-kB) (28). NF-kB is an important transcription factor that controls the expression of proinflammatory genes that can be activated by ROS (29). Nrf2 and NF-kB have been suggested as key molecules that modulate cellular redox status and the regulation of inflammation and stress response (30). The absence of Nrf2 can exacerbate NF-kB activity leading to increased cytokine production. In contrast, NF-KB can modulate Nrf2 transcription and activity and positively and negatively affect target gene expression. (30). Mitogen-activated protein kinases (MAPKs), including extracellular signal-regulated kinase (ERK), p38, and Jun NH2-terminal kinase (JNK), have been reported to be indispensable at all stages of spermatogenesis events, including spermatozoa maturation and sperm motility (31, 32). It has been observed that p38 MAPK and NF-kB increase in testicular oxidative stress condition (33, 34).

In light of the studies, the effect of thymoquinone on the NRF2/NF-kB/MAPK pathway in testicular damage induced by Methotrexate was investigated with this study. In addition, testicular tissue was evaluated histopathologically and biochemically. Serums were also assessed biochemically.

## Materials and methods

***Animals***

This study was planned in Yozgat Bozok University Faculty of Medicine, Department of Histology-Embryology, in line with the approval of Erciyes University Experimental Animals Local Ethics Committee. All procedures were conducted in accordance with the Universal Declaration of Animal Rights, with the approval of the Erciyes University Experimental Animals Ethics Committee (Date: 03.02.2021, Decision no: 21/36). The experimental phase of the research was carried out at Erciyes University Experimental Research Application and Research Center (DEKAM). In this study, 40 Wistar albino type 8-week-old adult male rats of 150-200 g, produced in DEKAM, were used. The rats housed in cages were kept in a light/dark environment for 12 hr in the normal order of the day at 21 ^°^C, and their water and nutritional needs were provided. Experimental groups were formed by weighing the subjects and bringing them together so that their weights were close to each other.

There are 5 groups in total in the experiment. Each group was formed with 8 rats. Intraperitoneal saline was administered to the control group (n:8) for 10 days. Intraperitoneal olive oil was applied to the olive oil group (n:8) for 10 days. In the methotrexate group [n:8], a single dose of 20 mg/kg MTX (500 mg/20 ml, Koçak Farma, Turkey) was administered intraperitoneally on the first day of the experiment (35). Since methotrexate would be taken in liquid form, no solvent was used. To Thymoquinone Group (n:8) 10 mg/kg THQ (274666-5G, purity-98%, Sigma--Aldrich Co., St Louis, MO, USA) IP was applied for 10 days (36). MTX+THQ group (n:8) MTX: (20 mg/kg 1st-day single dose intraperitoneally); THQ: 10 mg/kg IP administered for 10 days. The Olive oil group was formed because THQ is soluble in olive oil. Therefore, the THQ group was administered IP. Since we applied it as a solvent, olive oil was also administered IP on the 10th day of the experiment. Two hours after the last injection, the rats were killed under high-dose ketamine+xylazine anesthesia.

***Spermiogram analysis***

After the rats were sacrificed, testes and epididymis weights were measured. Then, semen was obtained from the epididymis. Counting was done by dropping semen samples in hand into the makler camera. Counts were made under the microscope and data were recorded.

***Histological procedure***

Testicular tissues taken at the end of the experiment were first fixed in 10% formaldehyde solution for 4 hr and then in Boin solution. After fixation, the tissues were dehydrated by passing through a series of increasing grades of alcohol [50%, 70%, 80%, 96%, and 100%]. Tissues cleared with xylene were embedded in paraffin. Hematoxylin-Eosin [H-E] staining was applied to 5-6 μm thick sections to be taken from paraffin blocks, and histopathological changes in the testicular tissue were determined (36, 37). Histopathological findings will be based on Johnsen’s testicular biopsy score (Table 1)(37). In addition, the differences between the groups were determined by measuring the tubular diameter. Images were taken under the Olympus BX53 microscope.

***Immunohistochemistry***

The Avidin biotin peroxidase method was used to determine the difference in NRF2, HO-1, NFKB, and p38 expression in testis sections. Paraffin sections were incubated in an oven and then dewaxed in xylene. For antigen retrieval, 10% citrate buffer was applied in the microwave for 5 min at 600 w and then allowed to cool at room temperature for 10 min. Sections washed with phosphate buffer [PBS] were treated with 3% hydrogen peroxide (HO) for 12 min to inhibit endogenous peroxidase activity. It was washed again with PBS 3 times for 5 min. A staining kit (Lab Vision, Ultra Vision Detection System Large Volume, Anti-Polyvalent Thermo Scientific HRP) was used for the next steps. The serum block was distilled and kept at room temperature for 10 min. NRF2, HO-1, NFKB, and p38 antibodies were prepared and kept at +4 ^°^C for one night. Sections from antibodies were washed for 2x5 min with PBS. It was kept in the secondary antibody for 10 min at room temperature and then washed again with PBS for 2X5 min. HRP was treated with streptavidin for 10 min at room temperature. After rewashing, it was treated with the kit diaminobenzidine (DAB) for 1.5 min to make its immunoreactivity visible. After passing through an ascending series of alcohol and xylene, it was closed with entellan (36). Scoring was done for the immunoreactivity of each antibody. Measurements were made from 50 different areas for each group.

***Biochemical analysis***

Testicular tissues and blood were obtained from rats. The obtained sera and tissues were raised to -80 ^°^C. Tissues were homogenized before the study. Then centrifugation was applied and the supernatants were transferred to Eppendorf tubes. Superoxide dismutase (SOD)(Cat. No: 201-11-016972, Sun Red Biological Technology), Catalase (CAT) (Cat. No: 201-11-5106, Sun Red Biological Technology), Glutathione peroxidase (GPx) according to ELISA method ) (Cat. No: 201-11-170506, Sun Red Biological Technology), Malondialdehyde (MDA) (Cat. No: 201-11-015706, Sun Red Biological Technology), Tumor necrosis factor-alpha (TNF-alpha) (Cat. .No: 201-11-076506, Sun Red Biological Technology), Interleukin 1 beta (IL1- beta) (Cat. No: 201-11-0108, Sun Red Biological Technology), Interleukin 6 (IL-6) (Cat. No: 201-11-0136, Sun Red Biological Technology), Testosterone (Cat. No: 201-11-5126, Sun Red Biological Technology), Follicle stimulating hormone (FSH) (Cat. No: 201-11-0183, Sun Red Biological Technology). These kits were used to measure the amount of Red Biological Technology) and Luteinizing hormone (LH) (Cat. No: 201-11-2119, Sun Red Biological Technology). Measurements were made at 450 nm on the ELISA reader.

***Statistical analysis***

The Kolmogorov-Smirnov test was used to determine the normal distribution of the data. One-way analysis of variance and *post hoc* Tukey test were used to determine the differences between groups. Results are presented as mean±standard deviation [SD]. GraphPad Prism 8.0 software was used for statistical analysis. *P*<0.05 was considered statistically significant.

## Results

***Testis-epididymis weights and spermiogram results***

**Figure 1:**
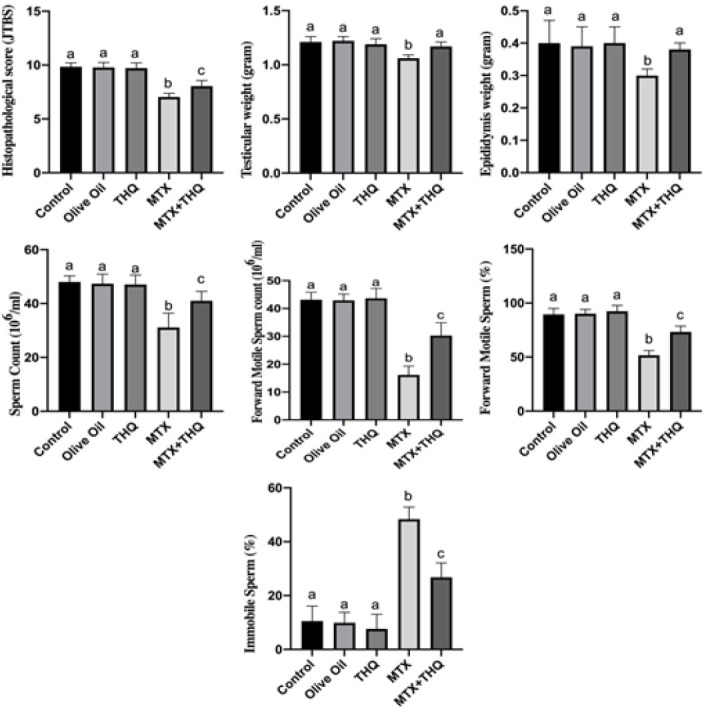
Results of Johnsen Testicular Biopsy Scoring, Testis-epididymis weight, and spermiogram results

Testicular and epididymis weight results and spermiogram results are shown in Figure 1. It was observed that testes and epididymis weights in the MTX group were statistically significantly decreased compared to the control group. In the MTX+THQ group, these weights were found to be close to the control group. Sperm count, advanced motile sperm count, and percentage of advanced motile sperm were found to be statistically significantly decreased in the MTX group compared to the control group. In the MTX+THQ group, these values were found to be close to the control group.

***Histopathological results***

**Figure 2:**
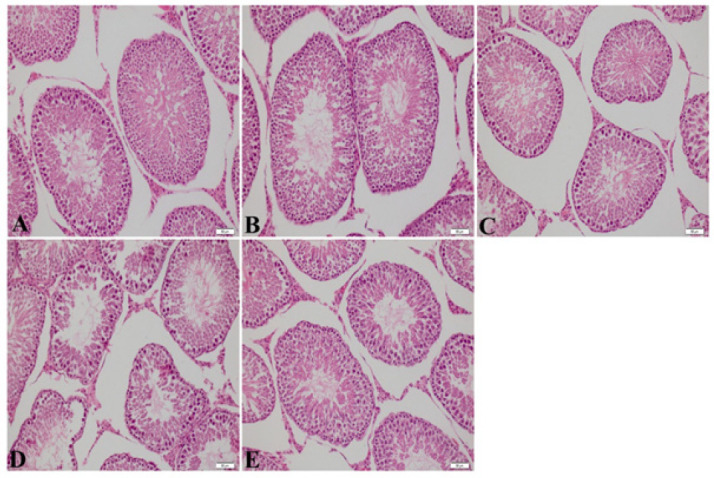
Testis Hematoxylin & Eosin Staining image

Hematoxylin & eosin staining images are shown in Figure 2. As a result of staining, a healthy structure was observed in the testicular tissue histologically in the Control, olive oil, and thymoquinone groups. In the methotrexate group, shedding of the seminiferous tubule epithelium in the testis tissue and a decrease in the spermatogenic series were observed. In the Methotrexate+Thymoquinone group, there was no such damage, and a structure close to the control group was observed (Figure 1).

***Immunohistochemical results***

**Figure 3:**
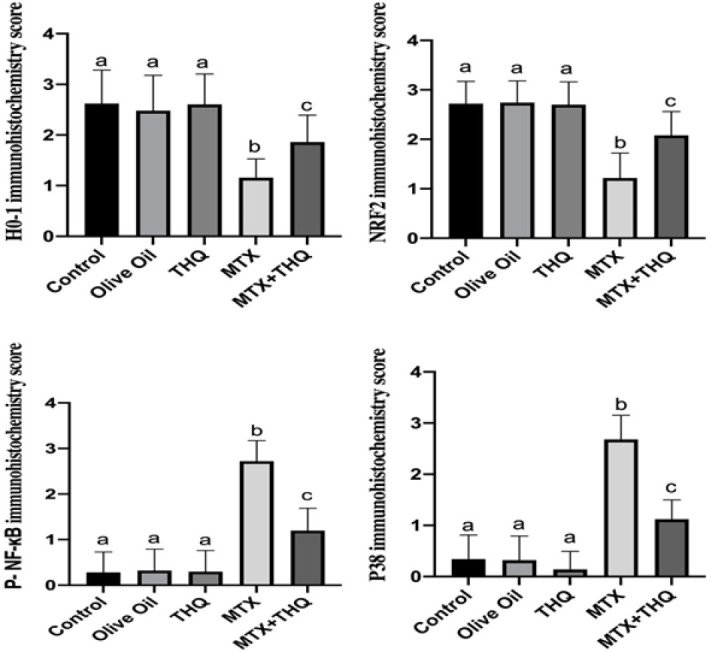
Testes immunohistochemistry results

**Figure 4:**
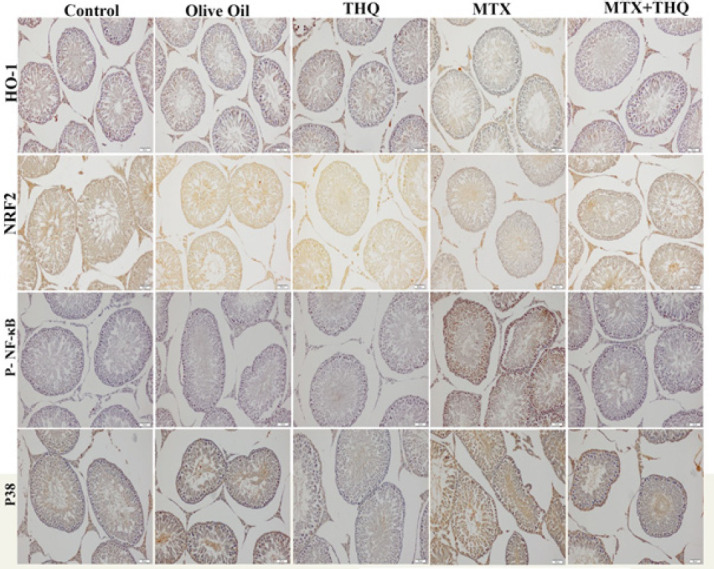
Testis NRF2, HO-1, NFKB, and p38 immunohistochemistry images

Testicular NRF2, HO-1, NFKB, and p38 immunohistochemistry results are shown in Figure 3 and Figure 4. NRF2 and HO-1 levels were found to be statistically significantly decreased in the MTX group compared to the control group. In the MTX+THQ group, these values were found to be close to the control group. NFKB and p38 levels were statistically significantly increased in the MTX group compared to the control group. In the MTX+THQ group, these values were found to be close to the control group.

***Biochemistry results***

**Figure 5:**
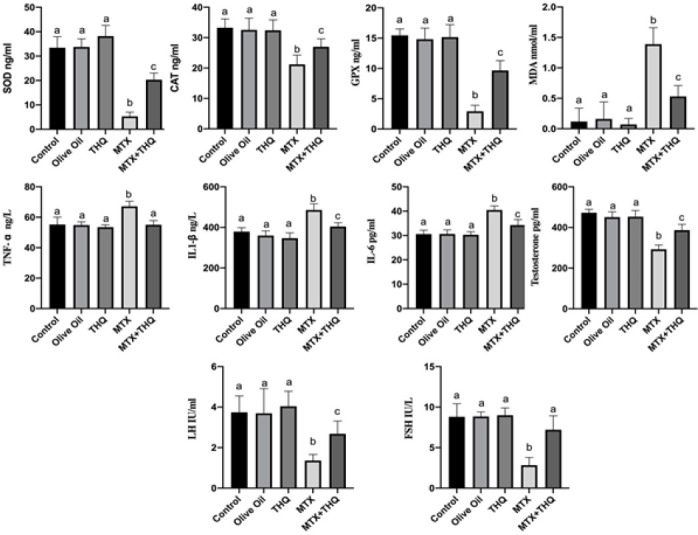
Testicular tissue biochemistry results (Antioxidant, inflammation and testosterone levels)

**Figure 6:**
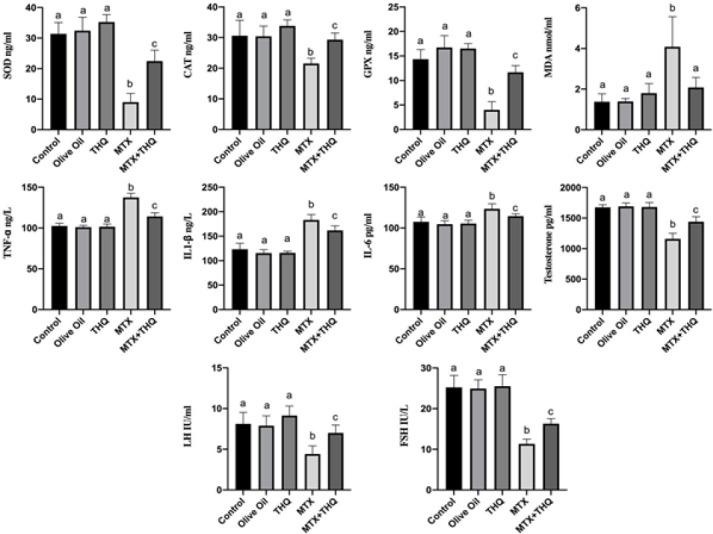
Serum biochemistry results (Antioxidant, inflammation and testosterone levels)

Testicular tissue and serum SOD, CAT, GPx, MDA, TNF-α, IL1-β, IL-6, testosterone, LH, and FSH results are shown in Figures 5 and 6. When the oxidant/antioxidant values were examined, it was observed that SOD, CAT, and GPx levels in both tissue and serum results were statistically significantly decreased in the MTX group compared to the control group. In the MTX+THQ group, it was observed that antioxidant levels increased and SOD, CAT, and GPx values increased significantly. When the inflammatory values were examined, it was observed that the levels of TNF-α, IL1-β, and IL-6 were statistically significantly increased in the MTX group compared to the control group. In the MTX+THQ group, on the other hand, it was observed that the inflammatory level decreased and TNF- α, IL1-β, and IL-6 values were significantly decreased. When hormone levels were evaluated, it was observed that testosterone, LH, and FSH levels were statistically significantly decreased in the MTX group compared to the control group. In the MTX+THQ group, however, this hormone level increased and testosterone, LH, and FSH values increased significantly.

## Discussion

Testicular damage is usually caused by pathological conditions in testicular structure and function. Toxic damage to the testis occurs as a result of the accumulation of free oxygen radicals (38). Methotrexate [MTX], a classical antifolate, is one of the most widely used and studied anticancer agents (38). However, the side effects of MTX limit its use. MTX causes testicular damage, especially in the male genital system (38). To reduce these side effects, it will be important to use substances that will reduce or prevent the harmful effects of MTX alongside the treatment. In recent studies, it has been observed that the antioxidant Thymoquinone [THQ] reduces MTX-induced germ cell damage, intestinal damage, and kidney damage (39-41). In the present study, we investigated the protective effect of THQ in MTX-induced testicular damage. 

It has been shown that significant reductions in testicular and epididymis weights occur in testis damage caused by MTX (38). It has also been shown in previous studies that MTX significantly reduces sperm parameters (12, 13). The current study is in agreement with previous studies indicating that MTX significantly reduced testicular and epididymis weights, as well as sperm parameters. This deterioration in sperm parameters will also contribute to the increase of oxidative stress. Significant improvements were observed in testicular and epididymis weight and sperm parameters [sperm count, motility, forward motility] with the use of THQ. Improvements in these sperm parameters suggest that THQ may be responsible due to its antioxidant properties (23).

MTX affects testicular cell number and dynamics. MTX-treated rats have tubular atrophy in their testicles with a decrease in germ cell count (15). In our study, a significant decrease and cell damage occurred in the spermatogenic series cells located in the seminiferous tubule epithelium in the MTX group. It was found that the damage to this spermatogenic series was significantly reduced in the MTX+THQ group. It is seen that gametogenesis is decreased in MTX-treated groups, while this gametogenesis is functional and recovers with a healthy appearance in THQ-treated groups. These results are in line with previous studies showing that THQ reduces seminiferous tubule damage (39, 42).

ROS are induced by drugs and toxic substances. As a result, oxidative damage occurs (25). In recent years, the role of the transcription factor NF-E2-related factor 2 [Nrf2] in regulating oxidative stress has received increasing attention. NRF2 is an important nuclear transcription factor that exerts its capacity to protect cells from oxidative stress (43) Nrf2, an important transcription factor, regulates the oxidative stress of cells and also plays an important function in maintaining intracellular redox homeostasis. Nrf2 can induce the expression of antioxidant protein. It can reduce cell damage caused by ROS and maintain the body’s redox homeostasis. Under normal physiological conditions, Nrf2 resides in the cytoplasm, binds with Keap1, and maintains Nrf2 at a low level. When receiving external stimuli, Nrf2 dissociates from Keap1, and after Nrf2 is transferred to the nucleus, it binds to the promoter region, activates Heme oxygenase-1 (HO1), and exerts an anti-inflammatory effect (44-47). HO-1 and its products exert beneficial effects through protection against oxidative damage, regulation of apoptosis, modulation of inflammation, and contribution to angiogenesis (48). In our study, MTX administration significantly reduced Nrf2 and HO-1 levels. The effect of MTX on the Nrf2/HO-1 signaling pathway is similar to previous studies (41, 49). Potentially reversed with the THQ implementation. Nrf2 plays a crucial role in regulating basal and inducible expressions of several cytoprotective and antioxidant genes that can counteract oxidative stress. The increased activity of the antioxidant defense system by THQ is directly attributable to its ability to positively regulate the Nrf2/HO-1 signaling pathway, matching this feature of THQ with previous studies (50, 51). Nrf2 and HO-1 can directly inhibit NF-kB signaling and proinflammatory cytokines and regulate the inflammatory cascade (49).

The p38 MAPK pathway has a vital role in the activation and induction of NF-kB, and the MAPK/NF-kB signaling pathway is a strategic regulator of inflammatory processes and acts as a regulator of inflammation in diseases (52). In this study, we proved that MTX significantly increased the levels of inflammatory mediators such as P38 MAPK and NF-kB in the testis, while the application of THQ significantly decreased the expression of all these parameters in the testis. Inflammation decreased in our study, as in the studies of diabetic testicular damage(53), neurotoxicity(54), and hypothyroidism-induced testicular damage with THQ (55). 

MTX has been reported to increase ROS production. MTX increases oxidative stress in induced testicular damage (16). MTX disrupts the antioxidant system (17). Oxidative stress, which causes testicular damage, develops due to the increase in ROS (18). Excessive production of ROS increases lipid peroxidation (24). In our study, it was observed that the level of antioxidant enzymes such as SOD, CAT, and GPx decreased significantly in testicular tissue and serum with the application of MTX. It has been observed that these decreased parameters improved with THQ application, similar to previous studies (53, 55). At the same time, it was observed that while MTX significantly increased the MDA level in testicular tissue and serum, THQ application reduced this lipid damage. The effect of THQ that increases this antioxidant capacity and reduces lipid damage has been shown in previous studies in the literature (53, 55). Excess ROS can also activate nuclear factor-kappa B (NF-kB) and the release of proinflammatory cytokines, triggering mitochondrial dysfunction and apoptosis. NF-kB activates the expression of inflammatory mediators (56). The increase in NF-kB expression by MTX led to an increase in the expression of inflammatory mediators (TNF-α, IL1-β, and IL6), which is in agreement with the studies performed (38, 49). We showed that the expression of these inflammatory mediators (TNF-α, IL1-β, and IL6) increased with THQ administration. The level of Inflammatory mediators was also decreased as THQ inhibited NF-kB/P38 expression. The anti-inflammatory effect of THQ has been demonstrated in different studies (20, 55).

MTX-induced testicular damage in rats is generally associated with spermatogenic damage, germ cell apoptosis, Leydig cell dysfunction, and testicular steroidogenic disorder (57). Our results showed that testosterone, LH, and FSH levels in serum and testicular tissue were significantly decreased in the MTX group. It has been confirmed by previous studies that the decrease in these parameters is due to both impaired Leydig cell function and the decrease in spermatogenic series and functional deterioration (58, 59). Testosterone, LH, and FSH levels that were decreased by MTX were restored with THQ administration. This confirms the effect of THQ in improving male infertility, similar to previous studies (60).

There was no significant difference between groups containing the same letter (a, b, c)THQ: Thymoquinone; MTX: Methotrexate

A. Control group, B. Olive oil group, C. THQ group, D. MTX group, E. MTX+THQ group. Image magnification X200.

THQ, Thymoquinone; MTX, Methotrexate

There was no significant difference between groups containing the same letter (a, b, c).

THQ, Thymoquinone; MTX, Methotrexate

Image magnification X200

THQ, Thymoquinone; MTX, Methotrexate

There was no significant difference between groups containing the same letter (a, b, c)

THQ, Thymoquinone; MTX, Methotrexate

There was no significant difference between groups containing the same letter (a, b, c)

THQ, Thymoquinone; MTX, Methotrexate

**Table 1:** Testicular biopsy score count (Johnsen score)

Score	Histological findings
1	Tubular section has no cells
2	There are only Sertoli cells
3	There are only spermatogonia as germ cells
4	Few (5/tubule) spermatocytes are present
5	There are many spermatocytes
6	Few (5/tubule) spermatids are present
7	Large number of spermatids without signs of differentiation
8	Late spermatids are present without mature spermatozoa
9	There are few (5/tubule) spermatozoa
10	There is complete spermatogenesis with a large number of spermatozoa

## Conclusion

According to these results, it is seen that important mechanisms such as morphological deterioration in seminiferous tubules, decrease in spermiogram, decrease in hormone levels that are important for male fertility, increase in inflammation, and decrease in antioxidant capacity resulting from MTX-induced testicular damage are significantly improved as a result of THQ application. We think that THQ will have an important place in infertility by researching more in terms of these features.

## Data Availability

We declare the availability of data related to this study.
